# Characteristics of patients with chronic hepatitis B infection in China: A retrospective claims database study

**DOI:** 10.1097/MD.0000000000036645

**Published:** 2024-02-16

**Authors:** Xing Meng, Iain A. Gillespie, Jane Dong, Yi Ning, Stuart Kendrick

**Affiliations:** a GSK, Institute for Infectious Diseases and Public Health, Beijing, China; b GSK, Epidemiology, Stevenage, UK; c School of Public Health, Hainan Medical University, Haikou, China; d GSK, Hepatology, Stevenage, UK; e Present address: Sinovac Biotech Co., Ltd, Clinical Research Department, Beijing, China.

**Keywords:** chronic hepatitis B, cirrhosis, healthcare costs, healthcare utilization, patient demographics, prevalence, treatment management

## Abstract

Chronic hepatitis B (CHB) infection affects approximately 90 million people in China, where there are profoundly unmet clinical and public health needs. This study evaluated patient demographics, disease progression, and treatment management using national administrative claims data. This retrospective, observational study used anonymized data from the China Health Insurance Research Association claims database (January 1–December 31, 2016); data that could not be validated, or from duplicate entries, were excluded. Patients were identified using the International Classification of Diseases, 10th Revision diagnostic code for CHB (B18.0 and B18.1), using keyword searches for “CHB or HBV” and free-text descriptions of CHB treatments including nucleos(t)ide analogues. Primary objectives included evaluation of: demographics and clinical characteristics of patients with CHB, overall and by presence or absence of cirrhosis and hospital tier; proportion of patients prescribed CHB treatment; and healthcare costs and utilization overall and by presence or absence of cirrhosis and hospital tier. Most identified patients with CHB were male, aged 25 to 65 years, resided in East China, and had employee health insurance. Cirrhosis was common (16.20%) and associated with male preponderance, older age, hepatitis C virus coinfection, and higher hospital care demands and costs. The most frequently visited hospitals were Tier III; patients visiting Tier III generally required more hospital care compared with those visiting Tier I/II hospitals. Only two-thirds of patients were prescribed antiviral therapy for CHB (most commonly nucleos(t)ide analogues). Results from this study highlight a substantial need to improve access to appropriate CHB treatment in China.

## 1. Introduction

Approximately 296 million people were living with chronic hepatitis B (CHB) globally in 2021.^[1]^ China has a large population of people living with CHB, estimated at one third of the global population with CHB in 2017.^[2]^ Although the initiation of hepatitis B virus (HBV) vaccination in 1992 reduced the rate of infection in the younger Chinese population, estimated hepatitis B surface antigen (HBsAg) seroprevalence in China overall (5.6%) remained higher than the global seroprevalence (3.2%) in 2022.^[3]^

Cirrhosis and hepatocellular carcinoma (HCC) represent major sequelae of CHB infection. Annually, approximately 2% to 10% of patients with CHB develop cirrhosis, of whom 3% to 5% progress to decompensated cirrhosis, associated with high morbidity and mortality and increased economic costs.^[4,5]^ In patients with CHB, the annual incidence of HCC is approximately 6 times higher for patients with compensated cirrhosis than for those without cirrhosis.^[4,5]^

Substantial clinical research has been conducted in China in different groups of patients with CHB. Clinical trials and post-approval studies in China are often conducted in tertiary or Tier III hospitals, which are large, state-owned provincial or municipal-level hospitals in urban areas.^[5,6]^ Therefore, the relevance of data arising from this setting to the wider population in China is uncertain; clinical practice, standards of care, and associated medical costs may differ in primary and secondary hospitals, as well as wider medical healthcare centers. Furthermore, real-world studies assessing CHB in China focused on specific regions,^[7–9]^ patient populations,^[10–12]^ or treatment approaches.^[11,13,14]^ For example, the China Kadoorie Biobank Collaborative Group assessed a large cohort of 0.5 million people in China, focusing on mortality or risk of chronic kidney disease.^[15,16]^ Consequently there is a need for comprehensive contemporary real-world data that describe patients in China with CHB in all geographies and in all healthcare settings.

This study therefore aimed to assess the distribution, demographic and clinical characteristics, associated treatment patterns, and healthcare utilization for patients with CHB in China using national administrative claims data from 2016. The population has also been described by cirrhosis status to identify patients in need of urgent medical care, both overall and by presence or absence of cirrhosis and also hospital level (Tier III or Tier I/II).

## 2. Materials and Methods

### 2.1. Data sources

This retrospective observational study was conducted using the China Health Insurance Research Association (CHIRA) database, the only national claims database in China. Data are collected annually from local health insurance bureaus, with consecutive inpatient and outpatient visit claims captured during a 1-year period; Chinese residents covered by China’s national health insurance program either through employee insurance or residential insurance (e.g., freelancers, juveniles) are captured.^[17,18]^ In 2016, >95% of the population were covered by public health insurance.^[19]^ The CHIRA database mainly consists of patients covered by insurance programs available predominantly in urban parts of China.^[17]^ Data from patients living in rural China, covered by the New Rural Cooperative Medical Scheme, were added to the CHIRA database in 2016; however, not all Medical Insurance Bureaus supplied patient data.

Data domains include demographics, hospital level (Tier III or Tier I/II), diagnoses, treatment, and financial cost.^[18]^ A sampling strategy is used to capture de-identified data annually from sampling cities (rural and urban areas) in a proportion of the insured population in each city (2% of municipalities and provincial capital cities; 5% of prefecture-level cities).^[17,18]^ Additional sampling is used to ensure appropriate age-specific distribution of medical use and treatment records over the calendar year, and all data from this selected sample are used to form the CHIRA database for the year.^[20]^ Longitudinal information on a given individual included in the database is not provided, as different individuals are selected each year; the study period was therefore 1 year.

The current study was conducted using anonymized data from the 2016 CHIRA claims database (January 1–December 31, 2016), based on data collected from sampling cities. Data that could not be validated, or from duplicate entries, were excluded. No additional data were collected from any other source.

The study sponsor did not have access to individual patient data that were already anonymized and received only summary tables.

### 2.2. Objectives

The primary objectives included ascertaining the proportion of patients diagnosed with CHB registered to the CHIRA database and describing demographic and clinical characteristics (age, gender, coinfection status, comorbidities, liver transplant status, pregnancy), overall and by presence or absence of cirrhosis and hospital tier. Other demographic variables included region, health insurance type, city level, and hospital type.

Primary objectives also included describing the proportion of patients prescribed CHB-related medication, associated healthcare costs (including aggregated treatment costs), and healthcare utilization (including hospital visits) for patients with CHB registered to the CHIRA database, overall and by presence/absence of cirrhosis and hospital tier.

Secondary objectives included describing patient characteristics, treatment patterns, costs, and healthcare utilization according to hospital tier.

### 2.3. Patient identification and data extraction

Patients eligible for inclusion were identified using the International Classification of Diseases, 10th Revision diagnostic code for CHB (B18.0 and B18.1), using keyword searches for ‘CHB or HBV’ and free-text descriptions of drugs in the treatment of CHB including nucleos(t)ide analogues (NAs); lamivudine (拉米夫定), telbivudine (替比夫定), adefovir dipivoxil (阿德福韦酯), entecavir (恩替卡韦), or tenofovir disoproxil fumarate (替诺福韦酯) prescribed with the diagnosis of hepatitis (肝炎). Patients were also required to be 18 to 70 years of age. In Chinese clinical practice, patients with HBV whose infection continues beyond the acute phase fall into 2 distinct groups (with unique ICD codes) based on clinical characteristics: patients who are HBsAg positive for ≥ 6 months with no abnormal liver function and no symptoms of hepatitis are considered to be HBV carriers, whereas patients with active signs of disease or abnormal liver tests are considered to have CHB.^[5]^ The current study included only CHB-diagnosed patients.

Patients were excluded based on criteria that could impact their CHB disease characteristics and/or treatment and hence challenge the interpretation of study findings. Those patients co-infected with other hepatitis viruses (A or E) were excluded, as were any patients with cancer. Patients with other causes of liver disease or those who had received a liver transplant were also excluded, as were patients with chronic kidney disease. Finally, women identified as pregnant on the basis of ICD codes were excluded.

The prevalence of diagnosed CHB infection was calculated as the number of patients diagnosed with CHB in relation to the number of enrollees in the 2016 CHIRA claims database. The number of visits included both inpatient and outpatient visits in 2016. Patient demographics, insurance plans, and city of residence were obtained using information provided at first CHB diagnosis. Comorbidities and complications were identified based on data from the whole year. Cancer type was collected as a mutually inclusive category, so these data are presented as number and percentage of instances without 95% confidence intervals (CI). CHB-related treatments included interferons (IFNs), NAs, and hepatic protective drugs. Traditional Chinese medicines were excluded. Treatment status was defined based on the date of the first CHB diagnostic code in 2016. Hospital visits were divided into outpatient and inpatient visits. The information on “associated cost” was categorized as “cost of medication,” which included all CHB-related medication, and “healthcare resources,” (all healthcare-related costs). These were recorded at CHB-related visits, defined as outpatient visits and inpatient visits concomitant with International Classification of Diseases, 10th Revision codes of CHB. The costs of CHB-related medication, which included 2 types of IFN and 5 types of NA, were collected by pharmacological grouping.

### 2.4. Statistical analysis

Relative frequencies and percentages were calculated for categorical data; means ± standard deviations (SD) and medians (interquartile range) were calculated for continuous data. A 95% CI was calculated for means and proportions as required. To assess the potential effect of vaccination policy, the prevalence of those born after the introduction of the vaccination policy (aged < 25 years) was compared with those born before; vaccine effectiveness was calculated as 1 minus the relative risk of infection in the former group versus the latter. For presentational clarity, lines of therapy accounting for < 1% were not included in the Sankey diagram.

### 2.5. Ethics

This study complied with all applicable laws regarding patient privacy. No direct patient contact or primary collection of individual human patient data occurred. Study results are in tabular form and presented as aggregate analyses that omit patient identification; therefore, informed consent, ethics committee, or IRB approval was not required.

## 3. Results

### 3.1. CHB diagnosed prevalence and age characteristics

The CHIRA database included 3,696,055 enrollees in 2016. Of these, 11,084 were patients with CHB, indicating an overall diagnosed prevalence of 0.30% (95% CI: 0.29–0.31). One patient had missing age data and was excluded from subsequent analyses, leaving 11,083 included patients.

**Figure 1.**
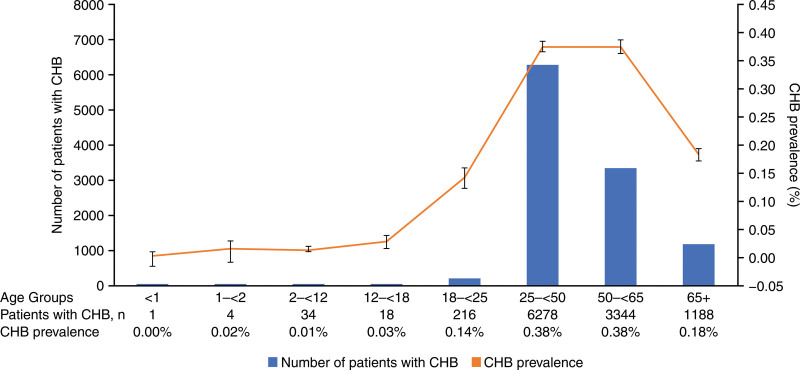
CHB prevalence in 2016 CHIRA database. Error bars represent 95% CI. CHB = chronic hepatitis B, CHIRA = China Health Insurance Research Association, CI = confidence interval.

Diagnosed CHB prevalence was low in children aged 12–<18 years (0.03 [95% CI: 0.01–0.04]) and higher in young adults aged 18–<25 years (0.14 [0.12–0.16]), with a substantial increase in adults aged 25–<50 years (0.38 [0.37–0.39]) and 50–<65 years (0.38 [0.36–0.39]). The prevalence of diagnosed CHB was comparatively lower in adults aged ≥ 65 years (0.18 [95% CI: 0.17–0.19]) than in those aged 25–<65 years (Fig. 1). The prevalence of CHB diagnosis in the < 25 years age group (0.06 [95% CI: 0.05–0.06]) was much lower than in the ≥ 25 years age group (0.34 [0.33–0.34]), corresponding to a vaccine effectiveness of 83.46 (95% CI: 81.35–85.33).

### 3.2. Baseline characteristics

Over two-thirds of patients with CHB were male (67.08 [95% CI: 67.08–67.09]), resided in East China (70.82 [70.81–70.83]), and had employee health insurance (85.85 [85.85–85.86]) (Table 1). Coinfections were rare: 0.03 (95% CI: 0.03–0.03) of patients with CHB were coinfected with human immunodeficiency virus (HIV), 0.6 (0.59–0.60) with hepatitis C virus (HCV), and 0.16 (0.16–0.16) with hepatitis delta virus; no patient was coinfected with both HIV and HCV. There were 999 patients with CHB who had cancer (9.01 [95% CI: 9.01–9.02]), including 112 and 396 instances of HCC (1.01%) and non-HCC (3.57%) liver cancer, respectively. Cirrhosis was common (16.20 [95% CI: 16.19–16.20]), but liver fibrosis was rare (2.84 [2.84–2.85]). Almost three-quarters (72.81 [95% CI: 72.79–72.83]) of patients with cirrhosis had compensated cirrhosis. Liver transplantations (0.19 [95% CI: 0.19–0.19]) and fatty liver disease (3.27 [3.26–3.27]) were rare.

**Table 1:** Demographic and clinical characteristics of patients with CHB.

Variable	Cirrhosis		Level of hospital			Total
	Yes	No	Tier III	Tier I & II	Crossed	N = 11,083
	N = 1795	N = 9288	N = 8174	N = 2521	N = 388	
Demographic						
Age, mean (SD)	54.46 (12.08)	45.02 (13.62)	45.85 (13.87)	48.29 (13.58)	50.05 (13.00)	46.55 (13.38)
Age group, n (%)						
<18	1 (0.06)	56 (0.6)	45 (0.55)	11 (0.44)	1 (0.26)	57 (0.52)
18–<25	3 (0.17)	213 (2.29)	163 (1.99)	51 (2.02)	2 (0.52)	216 (1.95)
25–<50	597 (33.26)	5681 (61.16)	4790 (58.60)	1300 (51.57)	188 (48.45)	6278 (56.65)
50–<65	821 (45.74)	2523 (27.16)	2353 (28.79)	846 (33.56)	145 (37.37)	3344 (30.17)
65+	373 (20.78)	815 (8.77)	823 (10.07)	313 (12.42)	52 (13.40)	1188 (10.72)
Gender, n (%)						
Male	1284 (71.53)	6151 (66.23)	5410 (66.19)	1752 (69.50)	273 (70.36)	7435 (67.08)
Female	511 (28.47)	3137 (33.77)	2764 (33.81)	769 (30.50)	115 (29.64)	3648 (32.92)
Region, n (%)						
East	1321 (73.59)	6528 (70.28)	5953 (72.83)	1574 (62.44)	322 (82.99)	7849 (70.82)
Middle	293 (16.32)	2149 (23.14)	1633 (19.98)	781 (30.98)	28 (7.22)	2442 (22.03)
West	181 (10.08)	611 (6.58)	588 (7.19)	166 (6.58)	38 (9.79)	792 (7.15)
City level, n (%)						
Municipality	515 (28.69)	2425 (26.11)	2461 (30.11)	354 (14.04)	125 (32.22)	2940 (26.53)
Provincial capital city	803 (44.74)	3254 (35.03)	3372 (41.25)	574 (22.77)	111 (28.61)	4057 (36.61)
Prefecture-level city	477 (26.57)	3609 (38.86)	2341 (28.64)	1593 (63.19)	152 (39.18)	4086 (36.87)
Insurance type, n (%)						
Resident health insurance	300 (16.71)	1268 (13.65)	937 (11.46)	587 (23.28)	44 (11.34)	1568 (14.15)
Employee health insurance	1495 (83.29)	8020 (86.35)	7237 (88.54)	1934 (76.72)	344 (88.66)	9515 (85.85)
Clinical, n (%)						
Coinfections						
HIV and HCV	0	0	0	0	0	0
HIV alone	0	3 (0.03)	3 (0.04)	0	0	3 (0.03)
HCV alone	25 (1.39)	41 (0.44)	53 (0.65)	11 (0.44)	2 (0.52)	66 (0.6)
HDV alone	N/A	N/A	N/A	N/A	N/A	18 (0.16)
Cancer*	547 (30.47)	452 (4.87)	805 (9.85)	130 (5.16)	64 (16.49)	999 (9.01)
Hematological cancer	195 (10.86)	190 (2.05)	319 (3.9)	42 (1.67)	24 (6.19)	385 (3.47)
HCC	74 (4.12)	38 (0.41)	93 (1.14)	14 (0.56)	5 (1.29)	112 (1.01)
Non-HCC liver cancer	255 (14.21)	141 (1.52)	316 (3.87)	51 (2.02)	29 (7.47)	396 (3.57)
Other solid-state cancer	23 (1.28)	83 (0.89)	77 (0.94)	23 (0.91)	6 (1.55)	106 (0.96)
Cirrhosis	1795 (100)	0	1349 (16.50)	330 (13.09)	116 (29.90)	1795 (16.20)
Compensated	1307 (72.81)	0	943 (11.54)	281 (11.15)	83 (21.39)	488 (4.40)
Decompensated	488 (27.19)	0	406 (4.97)	49 (1.94)	33 (8.51)	1307 (11.79)
Liver fibrosis	139 (7.74)	176 (1.89)	279 (3.41)	14 (0.56)	22 (5.67)	315 (2.84)
Liver transplant	3 (0.17)	18 (0.19)	19 (0.23)	1 (0.04)	1 (0.26)	21 (0.19)
Liver failure	82 (4.57)	45 (0.48)	91 (1.11)	23 (0.91)	13 (3.35)	127 (1.15)
Fatty liver disease	53 (2.95)	309 (3.33)	293 (3.58)	53 (2.10)	16 (4.12)	362 (3.27)
Hyperbilirubinemia	24 (1.34)	52 (0.56)	54 (0.66)	14 (0.56)	8 (2.06)	76 (0.69)
Hyperlipidemia	128 (7.13)	651 (7.01)	561 (6.86)	170 (6.74)	48 (12.37)	779 (7.03)
Cardiovascular diseases	466 (25.96)	1632 (17.57)	1523 (18.63)	457 (18.13)	118 (30.41)	2098 (18.93)
Cerebrovascular disease	141 (7.86)	580 (6.24)	499 (6.10)	188 (7.46)	34 (8.76)	721 (6. 51)
Hypertension	354 (19.72)	1312 (14.13)	1196 (14.63)	367 (14.56)	103 (26.55)	1666 (15.03)
Diabetes	240 (13.37)	663 (7.14)	645 (7.89)	197 (7.81)	61 (15.72)	903 (8.15)
CKD	40 (2.23)	224 (2.41)	203 (2.48)	46 (1.82)	15 (3.87)	264 (2.38)
Pregnancy	6 (0.33)	182 (1.96)	155 (1.90)	28 (1.11)	5 (1.29)	188 (1.70)

“Tier III” describes patients who visited Tier III hospitals only.

“Tier I/II” describes patients who visited Tier I/II hospitals only.

“Crossed” described patients who visited both Tier III hospitals and Tier I/II hospitals.

CHB = chronic hepatitis B, CKD = chronic kidney disease, HCC = hepatocellular carcinoma, HCV = hepatitis C virus, HDV = hepatitis D virus, HIV = human immunodeficiency virus, N/A = not available, SD = standard deviation.

* “Cancer” category is mutually inclusive, so presents number and percentage of instances, not of patients.

Compared with those without cirrhosis, patients with cirrhosis were older (mean age: 45.02 years [95% CI: 44.74–45.30] vs 54.46 years [53.90–55.02], respectively), more likely to be male (66.23 [66.22–66.24] vs 71.53 [71.51–71.55]), and more often coinfected with HCV (0.44 [0.44–0.44] vs 1.39 [1.39–1.40]). Cancer was more prevalent among patients with cirrhosis (30.47 [95% CI: 28.34–32.60]) than those without (4.87 [4.43–5.30]). Hepatocellular carcinoma was observed in 74 patients with cirrhosis (4.12%) and 38 without (0.41%); non-HCC liver cancers were observed in 255 (14.21%) cirrhotic and 141 (1.52%) non-cirrhotic patients. A numerical imbalance between cirrhotic and non-cirrhotic patients was also observed for hematological cancer (10.86% and 2.05%, respectively).

Almost three-quarters (73.75%) of patients visited Tier III hospitals only, more than one-fifth (22.75%) visited Tier I/II hospitals only, and a small proportion (3.50%) visited both hospital types. Compared with patients who visited Tier I/II hospitals, patients who visited Tier III hospitals were younger (mean age: 48.29 years [95% CI: 47.76–48.82] vs 45.85 years [45.55–46.15], respectively), more often resided in municipalities (14.04 [14.03–14.06] vs 30.11 [30.10–30.12]) or provincial capital cities (22.77 [22.75–22.79] vs 41.25 [41.24–41.26]), more often resided in Eastern regions of China (62.44 [62.42–62.45] vs 72.83 [72.82–72.84]), and were more likely to be covered by employee health insurance (76.72 [76.70–76.73] vs 88.54 [88.53–88.54]). Liver health was generally poorer in patients who visited Tier III hospitals, with several liver-related conditions (cirrhosis, liver failure, and hyperbilirubinemia) more prevalent in this group. Cancers were almost twice as prevalent in patients who visited Tier III hospitals (9.85 [95% CI: 9.20–10.49]) compared with patients who visited Tier I/II hospitals (5.16 [4.29–6.02]). The prevalence of other chronic comorbidities, such as cardiovascular disease, hypertension, and diabetes, was similar across both groups. For further information, see Table S1, Supplemental Digital Content, http://links.lww.com/MD/L438 which presents additional demographic and clinical characteristics.

### 3.3. Treatment

**Figure 2.**
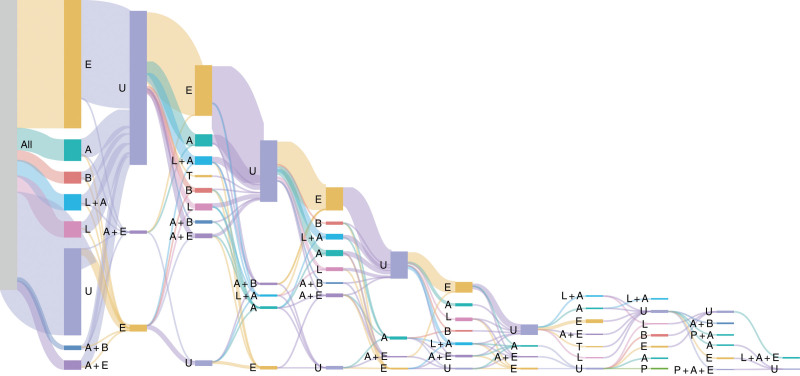
Treatment pattern of patients with CHB in 2016. The nodes (vertical bars) represent the order of treatments prescribed in the year, and the width of the links (horizontal connectors) represent the proportion of patients moving from one treatment state to another. Lines of therapy prescribed ≥ 1% are presented here. A = adefovir, B = telbivudine, CHB = chronic hepatitis B, E = entecavir, L = lamivudine, P = pegylated interferon, U = untreated.

Two-thirds of patients with CHB were prescribed anti-HBV treatments at index. For those prescribed anti-HBV treatment, the most common were NAs (97.37%), alone or in combination (Table 2). Few treated patients were prescribed IFNs, either alone (1.68%) or in combination with NAs (0.95%). A total of 3638 patients (32.83%) were not prescribed anti-HBV treatments, just over one-quarter of whom (26.66%) were prescribed other treatments, such as hepatic protective drugs and thymopeptide. The most common drug for patients receiving anti-HBV treatment was entecavir (66.00%), prescribed as a monotherapy or in combination with IFNs or other NAs. Anti-HBV therapy prescription patterns were captured monthly and are presented in a Sankey plot (Fig. 2). Contrary to expectations, patients appeared to cycle on and off NA therapy, suggesting bimonthly NA prescribing rather than monthly.

**Table 2:** Treatment characteristics for patients with CHB over a 12-month period in 2016.

Variable, n (%)	Cirrhosis		Level of hospital			Total
	Yes	No	Tier III	Tier I & II	Crossed	N = 11,083
	N = 1795	N = 9288	N = 8174	N = 2521	N = 388	
Treatment						
No antiviral treatment	356 (19.83)	3282 (35.34)	2708 (33.13)	812 (32.21)	118 (30.41)	3638 (32.83)
No treatment	226 (12.59)	2442 (26.29)	2008 (24.57)	572 (22.69)	88 (22.68)	2668 (24.07)
Other treatments*	130 (7.24)	840 (9.04)	700 (8.56)	240 (9.52)	30 (7.73)	970 (8.75)
Antiviral treatment	1439 (80.17)	6006 (64.66)	5466 (66.87)	1709 (67.79)	270 (69.59)	7445 (67.17)
NAs	1429 (79.61)	5820 (62.66)	5296 (64.79)	1686 (66.88)	267 (68.81)	7249 (65.41)
NA monotherapy	1287 (71.70)	4995 (53.78)	4564 (55.84)	1483 (58.83)	235 (60.57)	6282 (56.68)
ETV	1043 (58.11)	3486 (37.53)	3368 (41.20)	999 (39.63)	162 (41.75)	4529 (40.86)
TDF	2 (0.11)	45 (0.48)	35 (0.43)	11 (0.44)	1 (0.26)	47 (0.42)
LdT	34 (1.89)	355 (3.82)	329 (4.02)	52 (2.06)	8 (2.06)	389 (3.51)
LAM	91 (5.07)	477 (5.14)	339 (4.15)	199 (7.89)	30 (7.73)	568 (5.12)
ADV	117 (6.52)	632 (6.80)	493 (6.03)	222 (8.81)	34 (8.76)	749 (6.76)
NAs combination	142 (7.91)	825 (8.88)	732 (8.96)	203 (8.05)	32 (8.25)	967 (8.73)
LAM + ADV	70 (3.90)	463 (4.98)	393 (4.81)	123 (4.88)	17 (4.38)	533 (4.81)
ETV + ADV	47 (2.62)	252 (2.71)	223 (2.73)	66 (2.62)	10 (2.58)	299 (2.70)
LdT + ADV	16 (0.89)	89 (0.96)	98 (1.20)	3 (0.12)	4 (1.03)	105 (0.95)
Other NAs combination	9 (0.50)	21 (0.23)	18 (0.22)	11 (0.44)	1 (0.26)	30 (0.27)
IFN	5 (0.28)	120 (1.29)	108 (1.32)	15 (0.60)	2 (0.52)	125 (1.13)
IFN-α	2 (0.11)	33 (0.36)	24 (0.29)	10 (0.40)	1 (0.26)	35 (0.32)
PegIFN-α	3 (0.17)	87 (0.94)	84 (1.03)	5 (0.20)	1 (0.26)	90 (0.81)
IFN/NA combination	5 (0.28)	66 (0.71)	62 (0.76)	8 (0.32)	1 (0.26)	71 (0.64)
IFN + ETV	2 (0.11)	52 (0.56)	49 (0.60)	5 (0.20)	0	54 (0.49)
IFN + ADV	0	7 (0.08)	5 (0.06)	2 (0.08)	0	7 (0.06)
IFN + ETV + ADV	2 (0.11)	2 (0.02)	4 (0.05)	0	0	4 (0.04)
Other IFN/NA combination	1 (0.06)	5 (0.05)	4 (0.05)	1 (0.04)	1 (0.26)	6 (0.05)

“Tier III” describes patients who visited Tier III hospitals only.

“Tier I/II” describes patients who visited Tier I/II hospitals only.

“Crossed” described patients who visited both Tier III hospitals and Tier I/II hospitals.

ADV = adefovir, CHB = chronic hepatitis B, ETV = entecavir, IFN = interferon, LAM = lamivudine, LdT = telbivudine, NA = nucleos(t)ide analogue, pegIFN = pegylated interferon, TDF = tenofovir.

* Includes hepatic protective drugs, thymopeptide, and other treatments.

**Figure 3.**
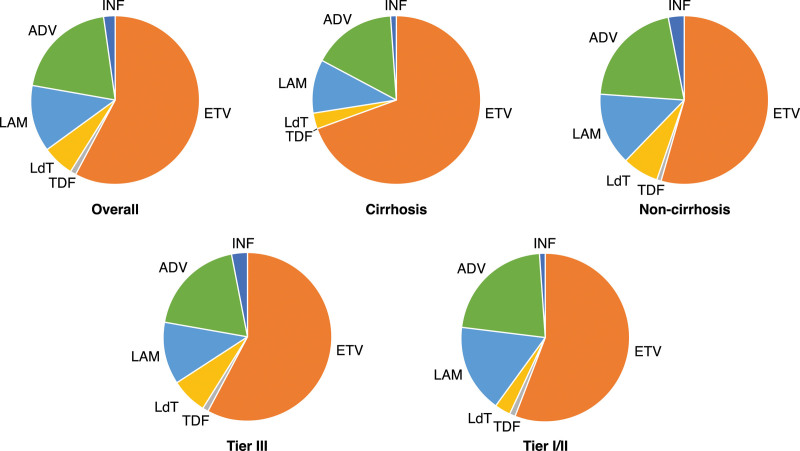
Market share of anti-HBV therapy overall and by cirrhosis status and hospital tier. “Tier III” describes patients who visited Tier III hospitals only. “Tier I/II” describes patients who visited Tier I/II hospitals only. ADV = adefovir, ETV = entecavir, HBV = hepatitis B virus, INF = interferon, LAM = lamivudine, LdT = telbivudine, TDF = tenofovir.

Overall, a higher proportion of patients with cirrhosis received anti-HBV treatment (80.17 [95% CI: 78.32–82.01]) compared with patients without cirrhosis (64.66 [63.69–65.64]). A higher proportion of patients with cirrhosis had NA prescriptions (79.61 [95% CI: 77.75–81.47]) compared with patients without (62.66 [61.68–63.65]). NA prescriptions consisted predominantly of entecavir for patients with cirrhosis but were more mixed for patients without cirrhosis (Table 2; Fig. 3).

The anti-HBV treatment rate was similar in patients who visited both Tier III and Tier I/II hospitals, with some exceptions. The NA entecavir was prescribed most commonly and in a similar proportion of patients in both hospital types. However, IFN prescriptions – alone or in combination with other therapies – varied between hospital type, with a 5-fold increase in pegylated IFN-α prescriptions (n = 84; 1.03% [95% CI: 0.81–1.25]) for patients who visited Tier III hospitals compared with those who visited Tier I/II hospitals (n = 5; 0.20 [exact 95% CI: 0.06–0.46]).

### 3.4. Healthcare utilization and cost

Almost three-quarters of patients with CHB (72.67%) attended ≥ 1 CHB-related outpatient visit, with a mean (SD) of 7.67 (7.15) outpatient visits per capita (Table 3). More than one-third (34.38%) of patients with active CHB including cirrhosis were hospitalized (admission reason was not captured in the CHIRA dataset), but this rarely occurred more than once, with a mean (SD) of 1.62 (1.74) inpatient visits per capita observed across the year.

**Table 3:** CHB-related cost and healthcare utilization of patients with CHB in 2016.

Variable	Cirrhosis		Level of hospital			Total
	Yes	No	Tier III	Tier I & II	Crossed	N = 11,083
	N = 1795	N = 9288	N = 8174	N = 2521	N = 388	
Total cost per capita (CNY), mean (SD) *	18,265 (28,624)	7024 (10,985)	9622 (16,884)	5512 (9104)	14,118 (22,633)	8845 (15,290)
Anti-HBV medicine						
Patients receiving treatment, n (%)	1700 (94.71)	7434 (80.04)	6724 (82.26)	2042 (81.00)	368 (94.85)	9134 (82.41)
Cost per capita (CNY), mean (SD)	4746 (5563)	4457 (5211)	4852 (5695)	3264 (3273)	5192 (5332)	4511 (5278)
Interferon						
Patients receiving treatment, n (%)	19 (1.06)	235 (2.53)	222 (2.72)	23 (0.91)	9 (2.32)	254 (2.29)
Cost per capita (CNY), mean (SD)	14,827 (15,948)	13,507 (13,961)	14,655 (14,534)	6171 (7525)	6709 (6563)	13,606 (14,112)
IFN-α						
Patients receiving treatment, n (%)	7 (0.39)	66 (0.71)	54 (0.66)	15 (0.60)	4 (1.03)	73 (0.66)
Cost per capita (CNY), mean (SD)	3197 (4099)	2802 (4272)	3199 (4653)	1370 (1860)	3504 (4114)	2840 (4258)
PegIFN-α						
Patients receiving treatment, n (%)	12 (0.67)	170 (1.83)	168 (2.06)	9 (0.36)	5 (1.29)	182 (1.64)
Cost per capita (CNY), mean (SD)	21,611 (16,453)	17,583 (14,220)	18,338 (14,717)	13,487 (6734)	9273 (7414)	17,849 (14,366)
NAs						
Patients receiving treatment, n (%)	1585 (88.30)	6452 (69.47)	5922 (72.45)	1774 (70.37)	341 (87.89)	8037 (72.52)
Cost per capita (CNY), mean (SD)	2922 (3275)	3690 (3497)	3783 (3685)	2686 (2553)	3720 (2964)	3539 (3454)
ETV						
Patients receiving treatment, n (%)	1287 (71.70)	4356 (46.90)	4232 (51.77)	1161 (46.05)	250 (64.43)	5643 (50.92)
Cost per capita (CNY), mean (SD)	2885 (3294)	3750 (3565)	3780 (3727)	2694 (2631)	3694 (2949)	3553 (3505)
TDF						
Patients receiving treatment, n (%)	10 (0.56)	123 (1.32)	97 (1.19)	28 (1.11)	8 (2.06)	133 (1.20)
Cost per capita (CNY), mean (SD)	1934 (1842)	1827 (2053)	1558 (1612)	2974 (3002)	1216 (859)	1835 (2039)
LdT						
Patients receiving treatment, n (%)	62 (3.45)	578 (6.22)	551 (6.74)	66 (2.62)	23 (5.93)	640 (5.77)
Cost per capita (CNY), mean (SD)	3069 (2523)	3197 (2607)	3235 (2651)	2860 (2255)	2917 (2194)	3185 (2599)
LAM						
Patients receiving treatment, n (%)	194 (10.81)	1091 (11.75)	844 (10.33)	372 (14.76)	69 (17.78)	1285 (11.59)
Cost per capita (CNY), mean (SD)	1591 (1624)	2180 (1599)	2207 (1653)	1917 (1527)	1616 (1474)	2091 (1603)
ADV						
Patients receiving treatment, n (%)	302 (16.82)	1684 (18.13)	1433 (17.53)	465 (18.45)	88 (22.68)	1986 (17.92)
Cost per capita (CNY), mean (SD)	1327 (1276)	1793 (1444)	1822 (1499)	1403 (1164)	1781 (1315)	1722 (1420)
Thymopeptide						
Patients receiving treatment, n (%)	274 (15.26)	355 (3.82)	424 (5.19)	164 (6.51)	41 (10.57)	629 (5.68)
Cost per capita (CNY), mean (SD)	2489 (3402)	2372 (3537)	2722 (3889)	1747 (1986)	2036 (3286)	2423 (3479)
Hepatic protective drugs†						
Patients receiving treatment, n (%)	1053 (58.66)	2807 (30.22)	2807 (34.34)	881 (34.95)	172 (44.33)	3860 (34.83)
Cost per capita (CNY), mean (SD)	2348 (3283)	1891 (2329)	2070 (2606)	1670 (2203)	2897 (4288)	2016 (2624)
Healthcare utilization‡						
Hospitalization						
N (%)	1038 (57.83)	2772 (29.84)	2731 (33.41)	930 (36.89)	149 (38.40)	3810 (34.38)
Frequency per capita	1.76 (1.47)	1.57 (1.83)	1.65 (1.90)	1.46 (1.17)	2.14 (1.38)	1.62 (1.74)
Cost per capita, mean (SD) (CNY)	25,570 (34,567)	12,965 (16,653)	18,024 (25,426)	10,064 (13,028)	26,153 (31,436)	16,399 (22,961)
Cost per hospitalization, mean (SD) (CNY)	14,904 (15,843)	9465 (10,635)	12,056 (13,147)	7258 (6603)	13,652 (21,207)	3810 (34.38)
Duration per hospitalization mean (SD) (Day)	14.78 (11.09)	15.53 (14.49)	15.54 (13.98)	14.87 (12.83)	14.28 (12.48)	–
Outpatient visit						
N (%)	1012 (56.38)	7042 (75.82)	5970 (73.04)	1743 (69.14)	341 (87.89)	8054 (72.67)
Frequency per capita	9.04 (6.94)	7.47 (7.18)	7.93 (7.15)	5.96 (6.39)	11.73 (8.71)	7.67 (7.15)
Cost per capita, mean (SD) (CNY)	6170 (7895)	4160 (4993)	4929 (6016)	2603 (2729)	4636 (4141)	4413 (5443)
Cost per outpatient visit, mean (SD) (CNY)	692 (620)	562 (457)	623 (512)	452 (368)	453 (91)	–

“Tier III” describes patients who visited Tier III hospitals only.

“Tier I/II” describes patients who visited Tier I/II hospitals only.

“Crossed” described patients who visited both Tier III hospitals and Tier I/II hospitals.

1 CNY = 0.1506 USD, 2016.

ADV = adefovir, CHB = chronic hepatitis B, CNY = Chinese yuan, ETV = entecavir, HBV = hepatitis B virus, IFN = interferon, LAM = lamivudine, LdT = telbivudine, NA = nucleos(t)ide analogue, pegIFN = pegylated interferon, SD = standard deviation, TDF = tenofovir, USD = United States dollars.

* Total cost of CHB-related visits, including all medicine and healthcare services, whether CHB relevant or not.

† Hepatic protective traditional Chinese drugs were not involved.

‡ Including all healthcare utilization information of CHB-related hospitalization and outpatient visits.

Patients with cirrhosis had a mean (SD) number of outpatients visits per capita of 9.04 (6.94) visits versus 7.47 (7.18) for those without cirrhosis. Mean (SD) outpatient cost per capita was 6170 (7895) Chinese yuan (CNY) (1 CNY = 0.1506 United States dollars, 2016) for patients with cirrhosis and 4160 (4993) CNY for patients without cirrhosis. Hospitalizations were experienced by 57.83% of patients with cirrhosis and 29.84% of those without cirrhosis; the mean (SD) frequency of admissions per capita was 1.76 (1.47) and 1.57 (1.83), respectively, and the mean (SD) hospitalization cost per capita was 25,570 (34,567) CNY and 12,965 (16,653) CNY, respectively. Consequently, the mean (SD) total cost per capita was 18,265 (28,624) CNY and 7024 (10,985) CNY for those with and without cirrhosis, respectively.

The mean (SD) number of outpatient visits for patients who visited Tier III hospitals and Tier I/II hospitals was 7.93 (7.15) and 5.96 (6.39), respectively. Hospitalizations occurred with 33.41% of patients who attended Tier III hospitals and 36.89% of patients who attended Tier I/II hospitals; mean (SD) frequency of hospital admissions was 1.65 (1.90) visits and 1.46 (1.17) visits, respectively. The mean (SD) days per hospitalization was 14.87 (12.83) days for Tier I/II and 15.54 (13.98) days for Tier III. The mean (SD) annual cost per hospitalization was 12,056 (13,147) CNY and 7258 (6603) CNY for patients attending Tier III and Tier I/II hospitals, respectively; mean hospitalization (SD) cost per capita was 18,024 (25,426) CNY and 10,064 (13,028) CNY, respectively. Overall mean (SD) cost per capita (includes mean of cost of outpatient visit and cost of hospitalization) was 9622 (16,884) CNY for patients who visited Tier III hospitals and 5512 (9104) CNY for those visiting Tier I/II hospitals.

## 4. Discussion

This study describes a cohort of Chinese patients with CHB according to their demographic and clinical characteristics, treatment patterns, and associated healthcare costs, as captured by a national administrative claims database. This was a nearly complete dataset, with only 1 missing age data point, demonstrating high-level data integrity and highlighting the benefits of using a national database to access demographic and clinical insight for a given disease.

Our study demonstrated an overall prevalence of CHB of 0.30% in the database population. Although diagnostic testing for HBsAg has been available since the early 1970s, a global study in 2022 showed the proportion of HBsAg-positive individuals who are accurately diagnosed remained low, at around 14%; despite this, the diagnostic rate in mainland China was high (24%).^[3]^ In the same study, estimated HBsAg prevalence in mainland China was 5.6%^[3]^; hence the proportion of diagnosed, HBsAg-positive individuals in the entire population of mainland China can be estimated to be 1.34% – a level far in excess of our current findings. This discrepancy may be partly due to specific characteristics of the CHIRA database. Our estimate was closer to, but distinct from, the 2016 prevalence of 0.14% reported in another study using the CHIRA database,^[18]^ highlighting the difference between population level seroprevalence (Polaris) and diagnosed CHB (CHIRA studies), which in itself is a subset of chronic HBV infection. The discrepancy in CHB prevalence estimated between the current and previous CHIRA study may be related to differences in the study eligibility criteria, the previous CHIRA study having fewer patients < 25 years of age (more likely to be vaccinated), and a lower proportion of patients ≥ 65 years of age (less likely to be vaccinated) compared with the present study.^[18]^

We report 83.5% less CHB prevalence in the < 25-year-old versus > 25-year-old age groups, demonstrating a substantial reduction in CHB infection in patients born after introduction of the national HBV vaccination program in China in 1992. This is consistent with earlier estimates of the reduction in the rate of HBsAg carriage following the implementation of the vaccination program,^[21,22]^ highlighting the importance of immunization. It is important to note that the CHIRA database selects on age only, but the preponderance for selection from urban areas may influence the underlying age distribution. The under-25 age group was underrepresented in CHIRA in 2016 compared with Chinese population estimates (Figure S1, Supplemental Digital Content, http://links.lww.com/MD/L440 shows the representativeness of the 2016 CHIRA population in the current study in relation to China census data), so some of the age-specific CHB pattern in younger age groups may be due to small sample size. The reported decline in CHB prevalence in the elderly population is possibly due to the additional complications of advanced disease, including cirrhosis and HCC, and reduced life expectancy.

Coinfection rates of HIV, HCV, and hepatitis delta virus were markedly low. The percentage of patients with HCV and HIV coinfection was similar to the national prevalence of HCV (0.7%)^[23]^ and HIV (<0.075%).^[24]^ However, most epidemiology research describes HBV or HCV coinfection rates in HIV-infected populations, with a lack of data on HIV coinfection in CHB or in HCV populations. In China, this may be due to the great difference between the infection rates of HIV and HBV in the general population, and the different major transmission routes – sexual transmission for HIV and maternal transmission for HBV.

Cirrhosis represents a significant part of disease progression following CHB infection and is associated with poor outcomes and high costs.^[4,5]^ This study found the prevalence of cirrhosis within the expected range and consistent with previous estimates.^[18]^ However, levels of fibrosis were lower than expected, perhaps due to a failure to adequately capture liver fibrosis using diagnostic codes, compounded by the limitations of liver biopsies or Fibroscans used in patients with CHB.^[25]^ Our study confirmed previous findings that patients with CHB and cirrhosis tend to be older^[26]^ and male.^[27]^ The observation of more cirrhosis in older patients was expected; as CHB progresses, cirrhosis develops with or without therapeutic intervention, resulting in more instances of CHB cirrhosis at a later age.^[28]^ The gender imbalance between patients with and without cirrhosis has been described previously and shown to be independent of lifestyle factors, suggesting that physiological differences exist that may account for this.^[29–31]^ Coinfection leads to more severe disease with CHB infection; consequently, the higher prevalence of HCV coinfection observed in patients with cirrhosis – even at the low levels reported in the current study – validates our findings. While the higher comorbidity burden observed in patients with cirrhosis compared with patients without cirrhosis has been described elsewhere,^[32]^ we find a 3% hematological cancer rate for patients with CHB; 10% and 2% reported for patients with and without cirrhosis, respectively. Anti-HBV treatment is recommended in patients undergoing chemotherapy or immunosuppressive therapy (e.g., rituximab) who are also HBsAg-positive. Patients with resolved hepatitis B, who are HBsAg-negative/hepatitis B core antibody-positive, are also at higher risk of reactivation on rituximab and hence require close monitoring for antiviral therapy.^[33]^ Therefore, it would be expected that HBV carriers with hematological cancer were more readily captured in this study, since patients with CHB were selected by diagnostic codes and medication. Overall, the greater healthcare utilization and costs for patients with cirrhosis compared with patients without cirrhosis suggest a need for earlier CHB treatment to minimize the complications associated with disease progression.

This study found that approximately two-thirds of patients with CHB were receiving anti-HBV therapy, which exceeds recent estimates of treatment uptake among patients with CHB in China^[34]^ and globally.^[1,35]^ While this study was conducted using the CHIRA database and is subject to specific limitations, which are discussed later, this may reflect the level of compliance with treatment guidelines by physicians or the accessibility of anti-HBV treatment through medical insurance coverage; it may also be indicative of the current level of patient education and treatment adherence. The reported bimonthly fluctuation in treatment pattern may signify poor treatment adherence. This observation may also be due to the national reimbursement system for patients in China, who must attend outpatient visits to access their monthly treatment and qualify for reimbursement, which requires taking time out of work. The high number of outpatient visits reported per capita (mean [SD]: 7.67 [7.15]) reflects this requirement. Alternatively, this bimonthly pattern may suggest patients are seeking their medication from sources other than their clinician, such as shops or the internet. Treatment with tenofovir was uncommon, received by only 0.42% of the total study population. This is likely a reflection that generic tenofovir was not available until 2017, after the data cutoff used in this study, and tenofovir was not added to the national reimbursement listing until May 2016, even though it was approved for use in China in October 2013. Recent evidence suggests that use of tenofovir in China is increasing, with a corresponding decrease in the use of first-generation NAs such as lamivudine, telbivudine, and adefovir dipivoxil.^[34]^

This study also aimed to compare patient characteristics and treatment patterns in Tier III versus Tier I/II hospitals. Our findings highlight key differences between patients with CHB who visited Tier III hospitals and those who visited Tier I/II hospitals. A greater number of patients who visited Tier III hospitals were reported to live in urban areas in the Eastern region of China compared with those who visited Tier I/II hospitals, reflecting an increased concentration of, and greater access to, Tier III hospitals in urban areas. Moreover, patients who visited Tier III hospitals were younger and presented with more advanced stages of CHB and CHB-related coinfections (e.g., fibrosis, cirrhosis, decompensated liver disease, and HCC) compared with patients who visited Tier I/II hospitals. These findings are expected because patients with more advanced stages of disease are more likely to seek medical care in Tier III hospitals. Higher healthcare utilization and costs for patients visiting Tier III versus Tier I/II hospitals were reflected by a higher associated cost of treating a larger group of patients with more advanced stages of disease. The different medications used, such as greater lamivudine and adefovir monotherapy prescribed in Tier I/II hospitals, may be due to differences in access to treatment, awareness of treatment guidelines, or affordability. These findings highlight the limitations of clinical trials, which are often carried out in Tier III hospitals, and thus may not provide an accurate depiction of disease treatment and progression for patients with CHB who only visit Tier I/II hospitals or who seek medical care elsewhere.

### 4.1. Limitations of the study

The findings from this study were based on an administrative claims database, which has potential for misclassification of disease or conditions, absence of data, or incorrect data. Due to our selection criteria, findings from our current study can only be generalized to patients with clinically managed CHB. Untreated and undiagnosed patients (including HBV carriers) are not captured.

Patient adherence to treatment could not be confirmed. In addition, we cannot rule out incorrect coding; for example, some patients diagnosed with “liver cancer” but not clearly differentiated as HCC could have been incorrectly coded as non-HCC liver cancer. The cross-sectional nature of this study also limits our ability to assess changes to treatment rates over time for CHB in China, which was taken at a single time point. Additionally, diagnosis rates and history of patients with CHB are not easily captured in this study. Therefore, additional longitudinal analyses are required to evaluate CHB clinical course and management across China.

## 5. Conclusion

This study represents a valuable contribution to understanding the burden and cost of CHB for China. We found that in 2016, diagnosed CHB infection was largely a disease of adulthood, likely due to the national HBV vaccination program introduced in 1992. Cirrhosis remains a significant factor in disease progression; our findings confirm that older patients, male patients, or HCV-coinfected patients are more likely to develop cirrhosis. We also found greater healthcare utilization and cost burden for patients with cirrhosis compared with those without, suggesting earlier treatment in CHB infection is required to minimize costs associated with disease progression. Nearly two-thirds of CHB patients in the claims system were on antiviral therapy with NAs (monotherapy or combination therapy), with IFNs prescribed less frequently, leaving a substantial proportion of untreated patients. This analysis also revealed high healthcare utilization and costs in Tier III versus Tier I/II hospitals, which may reflect the more advanced disease stage of patients in Tier III hospitals or differential access to and affordability of treatments.

## Author contributions

**Conceptualization:** Xing Meng, Iain Gillespie, Jane Dong, Yi Ning.

**Methodology:** Xing Meng, Iain Gillespie, Jane Dong, Yi Ning.

**Project administration:** Xing Meng, Jane Dong, Yi Ning.

**Writing – review & editing:** Xing Meng, Iain Gillespie, Jane Dong, Yi Ning, Stuart Kendrick.

## Supplementary Material




