# Supplementary material for: Characteristics of patients with chronic hepatitis B infection in China: A retrospective claims database study
Source: Medicine (Baltimore). 2024 Feb 16;103(7):e36645. doi: 10.1097/MD.0000000000036645 (PMC10869091; doi:10.1097/MD.0000000000036645)
Supplement: Supplementary file 1 [file medi-103-e36645-s001.docx]

## Table S1: Additional demographic and clinical characteristics of patients with CHB

| **Variable, n (%)** | **Cirrhosis** | | **Level of hospital** | | | **Total** |
| --- | --- | --- | --- | --- | --- | --- |
|  | **Yes** | **No** | **Tier III** | **Tier I & II** | **Crossed** | **N=11,083** |
|  | **N=1795** | **N=9288** | **N=8174** | **N=2521** | **N=388** |  |
| **Hospital type** |  |  |  |  |  |  |
| General | 1334 (74.32) | 6723 (72.38) | 5916 (72.38) | 1775 (70.41) | 366 (94.33) | 9395 (84.77) |
| Infectious diseases | 346 (19.28) | 1451 (15.62) | 1493 (18.27) | 248 (9.84) | 56 (14.43) | 1866 (16.84) |
| Other | 258 (14.37) | 1564 (16.84) | 1067 (13.05) | 603 (23.92) | 152 (39.18) | 4930 (44.48) |
| **Clinical** |  |  |  |  |  |  |
| **Depression** | 16 (0.89) | 107 (1.15) | 77 (0.94) | 39 (1.55) | 7 (1.80) | 123 (1.11) |
| **Myocardial infarction** | 5 (0.28) | 25 (0.27) | 16 (0.20) | 13 (0.52) | 1 (0.26) | 30 (0.27) |
| **Chronic kidney disease** | 40 (2.23) | 224 (2.41) | 203 (2.48) | 46 (1.82) | 15 (3.87) | 264 (2.38) |
| On dialysis | 3 (0.17) | 17 (0.18) | 19 (0.23) | 0 | 1 (0.26) | 20 (0.18) |

CHB, chronic hepatitis B.
